# Regulation of ovarian cancer by protein post-translational modifications

**DOI:** 10.3389/fonc.2024.1437953

**Published:** 2024-11-29

**Authors:** Qiugang Zhu, Huimin Zhou, Feiting Xie

**Affiliations:** ^1^ Department of Laboratory Medicine, Shangyu People’s Hospital of Shaoxing, Shaoxing University, Shaoxing, China; ^2^ Department of Laboratory Medicine, Wuxi Ninth People’s Hospital Affiliated to Soochow University, Wuxi, China; ^3^ Zhejiang Key Laboratory of Precision Diagnosis and Therapy for Major Gynecological Diseases, Women’s Hospital, Zhejiang University School of Medicine, Hangzhou, China

**Keywords:** ovarian cancer, post-translational modifications, pathogenesis, molecular mechanisms, therapeutic strategies

## Abstract

Ovarian cancer is one of the predominant gynecologic malignancies worldwide, ranking as the fifth leading cause of cancer-induced mortality among women globally. Post-translational modifications (PTMs) refer to the enzyme-catalyzed attachment of functional groups to proteins, thereby inducing structural and functional alterations. Recent evidence suggests that PTMs play multifaceted roles in the pathogenesis of ovarian cancer, influencing processes such as cell cycle, metabolism reprogramming, chemoresistance, and immune responses against cancer. Accordingly, a comprehensive understanding of the diverse PTMs in ovarian cancer is imperative for decoding the complex molecular mechanisms that drive cancer progression. This review discusses the latest developments in the study of protein PTMs in ovarian cancer and introduces pharmacological approaches that target these modifications as therapeutic strategies.

## 1 Introduction

Ovarian cancer (OC), the fifth most lethal malignancy among women globally, is particularly prevalent in women over 50 years old (1, 2). Due to the unique location of ovaries, their small size, tumor heterogeneity, and the absence of typical symptoms, OC is usually diagnosed at advanced stages frequently accompanied by extensive abdominal metastasis (3). Surgery and chemotherapy are the most common treatments, with radiotherapy and immunotherapy also being available (4). Unfortunately, there are still many patients who cannot benefit from these therapies. Recent findings have indicated that 37.5% (409/1092) of OC patients display tumor-based next-generation sequencing (tbNGS) results, with mutations most commonly in *TP53*, *PIK3CA*, and *NF1* (5). Proteomic and phosphoproteomic analyses have shown that Aurora kinases and Rho-associated kinase 1 (ROCK1) are major drivers of metastatic behaviors in epithelial ovarian cancer (EOC) cells, and phosphoproteomic reprogramming precedes proteomic changes that characterize spheroid readherence in EOC metastasis (6). Based on these findings, it is urgent and feasible to explore new biomarkers and targets for the early diagnosis and treatment of ovarian cancer.

**Figure 1:**
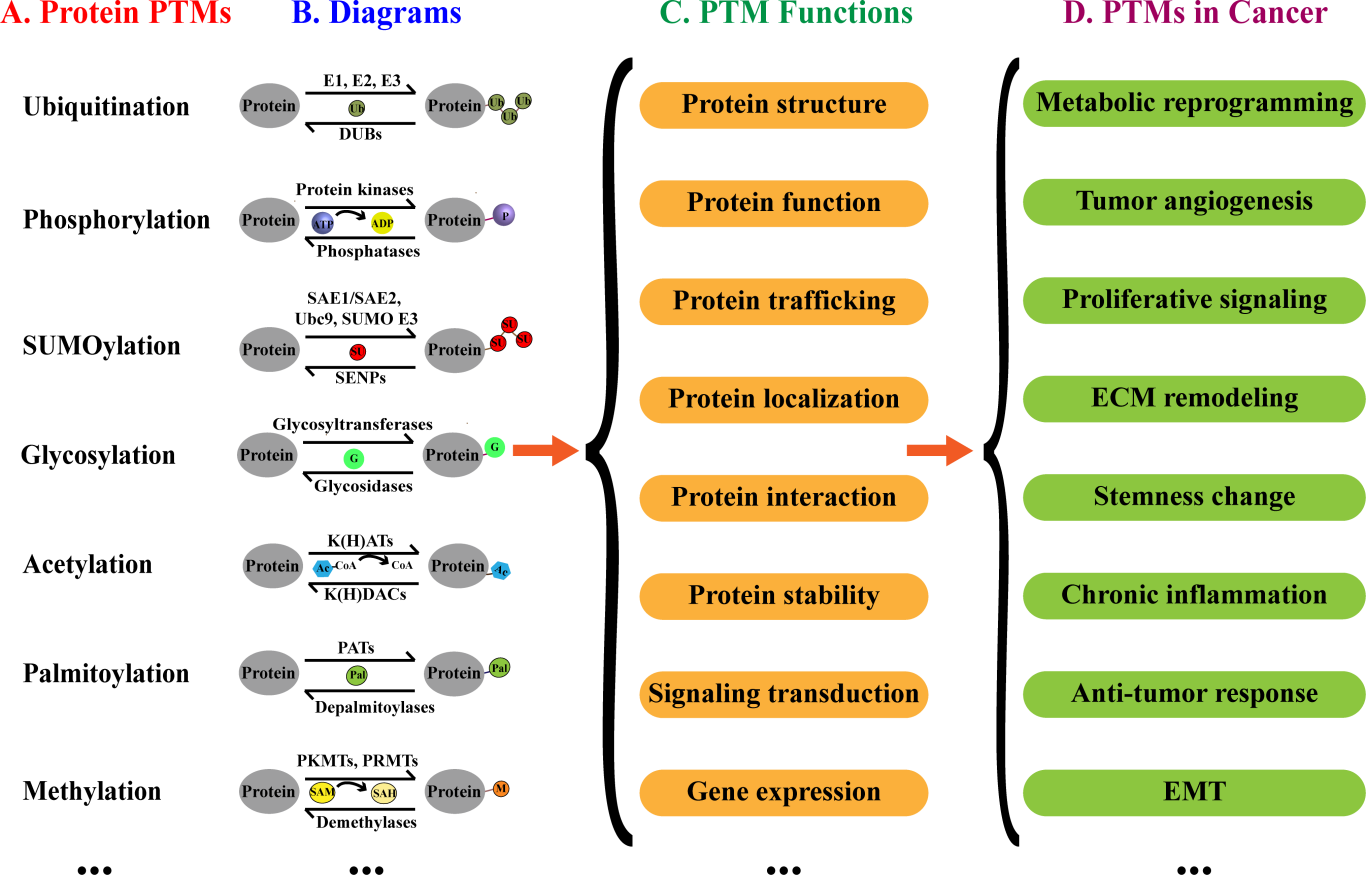
Protein PTMs, biological functions and implication in cancer. **(A)** Examples of PTMs. **(B)** Diagrams of PTMs. **(C)** Effects of PTMs on proteins. **(D)** Potential implications of PTMs in cancer.

Post-translational modifications (PTMs) refer to the modifications that occur after protein synthesis, playing a pivotal role in regulating biological processes (**Figure 1**) (7). Predominantly explored PTMs include ubiquitination, SUMOylation, acetylation, phosphorylation, palmitoylation, methylation, and glycosylation (8, 9). These modifications regulate cancer progression by affecting proliferation, metastasis, angiogenesis, and chemoresistance (10–12). PTMs can also regulate the cancer progression by downregulating anti-tumor immunity (13–15). Furthermore, PTMs play a crucial role in establishing and reshaping tumor microenvironment (TME), thereby influencing the efficacy of immunotherapy (16, 17).

CA125, a mucin (MUC) family glycoprotein, is markedly elevated in OC. The shed extracellular domains of CA125 can be used for clinical detection, and it has been reported that the cut-off of value for CA125 is 35 IU/mL, which includes 99% of healthy individuals (18). Additionally, CA125 levels are instrumental in monitoring therapeutic efficacy and prognosis (19). Clinically, elevated levels of PTM-related proteins such as F-box only protein 2/6 (FBXO 2/6), cullin 3 (CUL3), and lysine acetyltransferase 6A (KAT6A) correlated with the poor prognosis of OC (20–23). Clinical trials have demonstrated the good safety profile and efficacy of PTM modulators in OC, such as AKT inhibitors and WEE1 inhibitors (24, 25). Therefore, a comprehensive understanding of PTMs will offer valuable insights into the mechanisms underlying cancer progression and potentially facilitate the development of novel therapeutic strategies. In this review, we particularly outline the role of PTMs in the pathogenesis of OC, aiming to provide new perspectives for the future research. Meanwhile, we briefly introduce potential modulators of PTMs as therapeutic agents, review clinical trials utilizing PTM modifiers in OC.

## 2 PTMs in ovarian cancer

Reports have shown that PTMs orchestrate OC progression via different mechanisms including the regulation of oncogenic signaling, oncogenic cytokines, autophagy, cell adhesion, metabolic adaption, and drug resistance. In the following sections, we discuss the significant impacts of PTMs on OC progression (**Table 1**).

**Table 1:** The post-translational modifications (PTMs) of protein and their roles in ovarian cancer.

PTMs	Proteins	Mediators	Final effects	References
Ubiquitination	SUN2	FBXO2	Promotes the development of OC	(20)
	RNASET2	FBXO6	Promotes the development of OC	(21)
	hnRNPL	FBXO16	Suppresses the development of OC	(32)
	ARHGAP26	SMURF1	Promotes the development of OC	(34)
	BECN1	CUL3	Promotes the development of OC	(22)
	Smad4	GPBAR1	Promotes the development of OC	(35)
	HIF-1α	TRPM7 knockdown-induced AMPK activation	Suppresses the development of OC	(36)
	RPS3	SIAH1	Promotes chemosensitivity	(40)
Deubiquitination	PKM2	PSMD14	Promotes the development of OC	(37)
	STING	USP35	Promotes the development of OC	(39)
Phosphorylation	MLK3	CDK1	Suppresses the development of OC	(52)
		CDK2	Promotes the development of OC	
	FAK	RCP1	Promotes the development of OC	(54)
	MYLK	SIK2	Promotes the development of OC	(55)
	IRS4	FER	Promotes the development of OC	(57)
	PTEN	PKC	Promotes the development of OC	(58)
	STAT1	TG2 deficiency	Suppresses the development of OC	(56)
	FOXK2	PDK2	Promotes the development of OC	(59)
	EGFR	FGFR3	Promotes cisplatin resistance	(60)
	Cofilin-1	–	Promotes paclitaxel resistance	(61)
	TRIM37	PBK	Promotes PARP inhibitor resistance	(62)
	EZH2	AMPK	Suppresses the development of OC	(129)
Dephosphorylation	STAT3	TG2 deficiency	Promotes the development of OC	(56)
Glycosylation	CA125	–	Used as a disease marker	(68)
	HE4	–	Used as a disease marker, Establishes the suppressive TME, Promotes cisplatin resistance	(72–74)
	IgG	–	Used as disease markers	(75, 76)
	ITGα3	–	Used as disease markers	(77)
	CD82	MGAT3	Suppresses the metastasis of OC	(78)
SUMOylation	MEK1	–	Suppresses the development of OC	(81)
Acetylation	COP1	KATA6	Promotes the development of OC	(23)
	STAT3	–	Promotes the development of OC (via enhancing ARHI promoter methylation)	(87)
	Histone H4	HBO1	Enhances mechano-transduction pathways and membrane elasticity of OC cells	(91)
Deacetylation	HMGB1	SIRT1	Suppresses the development of OC	(89)
Palmitoylation	CLDN3	ZDHHC12	Promotes the development of OC	(96)
	MDH2	ZDHHC18	Promotes the development of OC	(97)
Neddylation	Cullin	–	Promotes the development of OC	(130)
Nitrosylation	Phosphofructokinase	NOS1	Promotes the development of OC	(131)
Methylation	Histone H3 (K79)	C/EBPβ-recruited methyltransferase DOT1L	Promotes platinum resistance	(101)
	Histone H3 (K4)	Histone-lysine N-methyltransferase 2D	Promotes the development of OC	(100)
	Histone H3 (K9)	G9A	Promotes the development of OC	(98, 132)
	BAF155	CARM1	Promotes the development of CARM1-expressing OC	(99)
Demethylation	Histone H3 (K4 and K27)	Nicotinamide N-methyltransferase	Promotes the development of OC	(102)

-:not mentioned.

### 2.1 Ubiquitination

**Figure 2:**
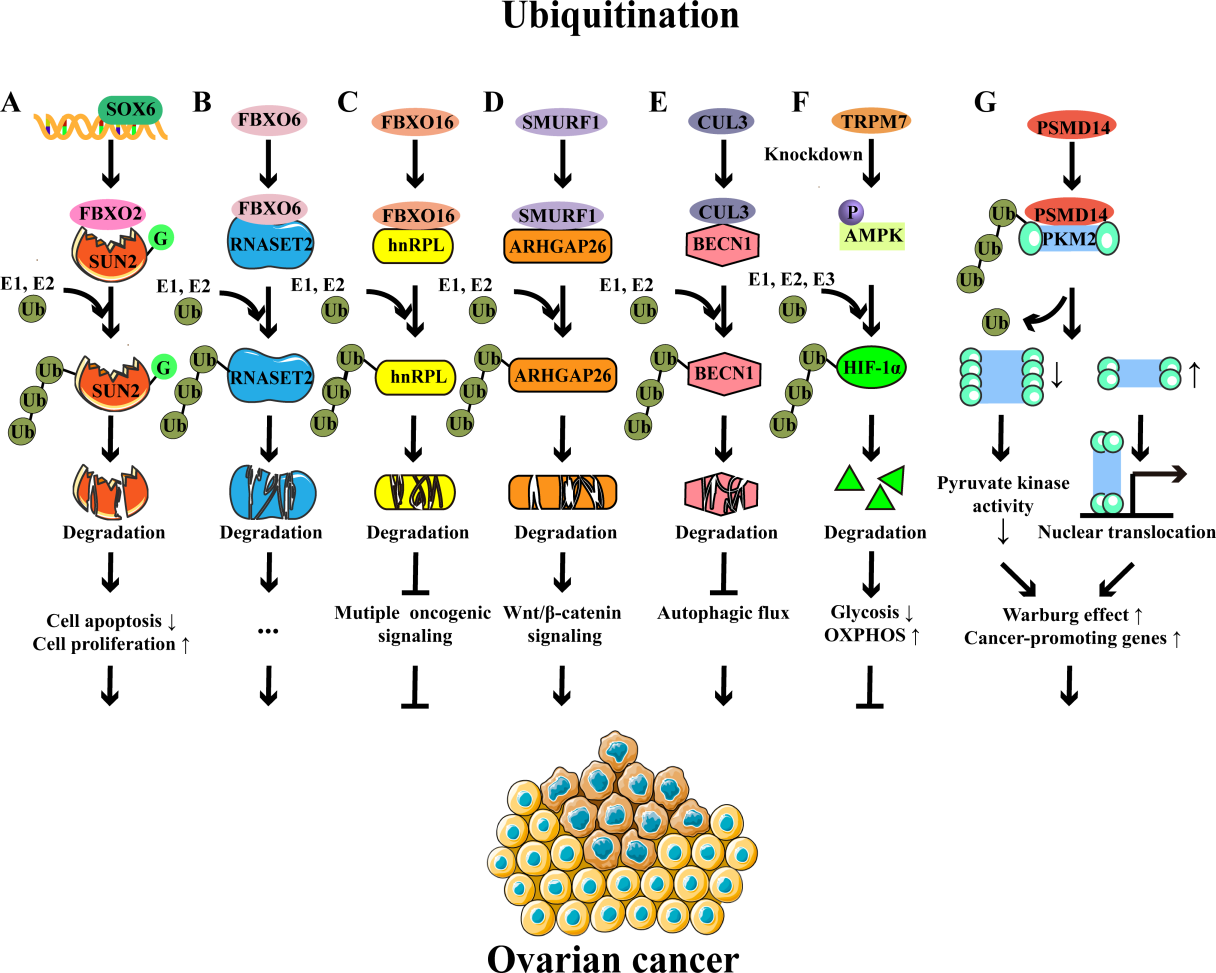
Examples of protein ubiquitination and their roles in ovarian cancer. **(A)** SOX6 promotes the expression of FBXO2, which functions as an E3 ligase to target the glycosylated SUN2 for ubiquitination-mediated degradation, thereby preventing cell apoptosis and promoting cell proliferation. **(B)** FBXO6-mediated RNASET2 ubiquitination and degradation facilitate the development of OC. **(C)** FBXO16-mediated hnRNPL ubiquitination and degradation inhibit multiple oncogenic signaling, thereby acting as a tumor suppressor in OC. **(D)** SMURF1-mediated ubiquitination and degradation of ARHGAP26 contribute to the progression of OC via the β-catenin pathway. **(E)** CUL3-mediated ubiquitination and degradation of BECN1 inhibit autophagic flux and subsequently lead to OC progression. **(F)** TRPM7 knockdown modulates glucose metabolic reprogramming to inhibit the OC growth by enhancing AMPK activation to promote HIF-1α degradation. **(G)** The deubiquitinase PSMD14 removes the K63-linked ubiquitin chains from PKM2, downregulates the ratio of PKM2 tetramers to dimers and monomers, and subsequently reduces pyruvate kinase activity and induces nuclear translocation of PKM2, contributing to glycolysis and oncogene expression.

Ubiquitination, a prevalent PTM, involves the covalent attachment of free ubiquitin to target proteins, thereby influencing their stability and biological functions and regulating a series of biological processes (26). Free ubiquitin is a small protein composed of 76 amino acids, featuring seven lysine (Lys) sites (K6, K11, K27, K29, K33, K48, and K63), one glycine (Gly) site at the C-terminal, and one methionine (Met) site at the N-terminal (27). Ubiquitination usually occurs at the Lys residue of target proteins, which is mediated by E1s, E2s, and E3s (27, 28). The E1 ubiquitin-activating enzyme utilizes ATP to activate ubiquitin through acyl-adenylation. This process involves the formation of ubiquitin-AMP intermediate, then ubiquitin is transferred to the active site of E1 through a thioester bond between the carboxy-terminal carboxyl group of ubiquitin and the sulfhydryl group of the E1 cysteine. Concurrently, AMP is released during this transfer (28, 29). Subsequently, the E2 ubiquitin-conjugating enzyme facilitates the transfer of ubiquitin from the E1-thio-Ub intermediate to E2, where E2 forms a thioester bond with the ubiquitin through its active site cysteine residue. Finally, the ubiquitination of the protein substrate can proceed through distinct mechanisms, contingent upon the specific E3 ubiquitin ligase involved. For members belonging to the homologous to E6AP C terminus (HECT) and really interesting new gene (RING)-between-RING (RBR) families, ubiquitin is initially delivered to the active site of the E3 ligase and then transferred to the protein. Conversely, for E3 ligases of the RING family, ubiquitin is directly shuttled from E2 to the protein, with the E3 ligase playing a facilitating role in this process (28, 29). Interestingly, ubiquitination is a reversible process that involves multiple types of deubiquitinases (DUBs) in the deubiquitination (30, 31). Importantly, both protein ubiquitination and deubiquitination have been implicated in the regulation of OC progression (**Figure 2**).

#### 2.1.1 Ubiquitination in cancer progression

The F-box protein family, comprising members such as FBXO2, FBXO6, and FBXO16, has been well-studied in OC (20, 21, 32). FBXO2 is an E3 ubiquitin ligase, and its upregulation is associated with the poor prognosis of OC patients (20). In the murine model, reduced tumor volume, size, and weight were observed in FBXO2-silenced A2780 cell implanted-mice compared to control mice (20). Mechanistically, FBXO2 interacted with glycosylated SAD1/UNC84 domain protein-2 (SUN2, a tumor suppressor protein) and then mediated its ubiquitination, accelerating the degradation of SUN2 and ultimately preventing the apoptosis of OC cells (20, 33). Similarly, FBXO6 was highly expressed in OC tissues, which promoted the proteasome-mediated degradation of ribonuclease T2 (RNASET2, a tumor suppressor protein) through K48-linked ubiquitination, consequently facilitating the proliferation, migration, and invasion of OC cells (21). Conversely, FBXO16 acted as a tumor suppressor in OC (32). Specifically, FBXO16 interacted with the C-terminal region of heterogeneous nuclear ribonucleoprotein L (hnRNPL), which resulted in ubiquitination-induced degradation of hnRNPL and subsequent oncogenic pathway inactivation, thereby restricting OC growth (32).

Smad ubiquitination regulatory factor 1 (SMURF1), an E3 ubiquitin ligase, was upregulated in OC patients. Research has shown that SMURF1 facilitates the proteasome-dependent degradation of Rho GTPase-activating protein 26 (ARHGAP26) through ubiquitination (34). The decreased level of ARHGAP26 effectively triggered the β-catenin signaling, as well as the expression of VEGF and MMP2/7, leading to the OC cell invasion and migration (34). CUL3 was also increased and correlated with the poor prognosis (22). CUL3 mediated the K48-linked ubiquitination of beclin 1 (BECN1), leading to its degradation and subsequent reduction in autophagy, ultimately contributing to tumor progression (22). A recent study has reported that G-protein-coupled bile acid receptor-1 (GPBAR1) is overexpressed in patients with serous ovarian cancer (SOC) and is significantly associated with the poor prognosis of OC (35). In this research, they have found that GPBAR1 promotes SOC development by inducing the ubiquitination of Smad4, accompanied by extracellular signal-regulated kinase (ERK) signaling activation (35).

Metabolic programs also influence the OC progression, which could be regulated by ubiquitination (36). Hypoxia-inducible factor-1α (HIF-1α), a crucial transcription factor governing cellular adaptability to hypoxic conditions, was regulated by transient receptor potential 7 (TRPM7) through ubiquitination (36). Mechanistically, the knockdown of TRPM7 promoted the phosphorylation of AMPK, which resulted in the enhanced ubiquitination-mediated degradation of HIF-1α, thereby causing a shift from glycolysis to oxidative phosphorylation and ultimately inhibiting the progression of OC (36). The deubiquitinase 26S proteasome non-ATPase regulatory subunit 14 (PSMD14) interacted with pyruvate kinase M2 isoform (PKM2) and cleaved K63‐linked ubiquitin chains from PKM2, leading to a decrease in the proportion of PKM2 tetramers relative to the oncogenic PKM2 dimer and monomer (37). These alterations reduced the pyruvate kinase activity of PKM2 and facilitated the nuclear translocation of PKM2, resulting in aerobic glycolysis and cancer‐promoting gene expression (37). Clinically, it has been confirmed that PSMD14 is highly expressed in OC tissues and is associated with the disease progression of OC (37). The knockdown of deubiquitinase Otubain 2 (OTUB2) has been reported to promote OC progression via mitochondrial metabolic reprogramming (38). This study revealed that the low expression of OTUB2 was correlated with the poor prognosis of OC patients, and depletion of OTUB2 promoted the progression of OC in the mouse model. Mechanistically, OTUB2 knockdown enhanced the K48-linked ubiquitination and degradation of sorting nexin 29 pseudogene 2 (SNX29P2), thereby protecting HIF-1α from degradation. The increased levels of HIF-1α promoted the carbonic anhydrase 9 (CA9) transcription to enhance the glycolysis, ultimately driving the progression of OC (38).

#### 2.1.2 Ubiquitination in drug-resistance

Several studies indicate that ubiquitination may also play crucial roles in chemoresistance (39, 40). Ribosomal protein S3 (RPS3) is a molecule that confers chemoresistance in EOC. A previous study demonstrated that seven in absentia homolog 1 (SIAH1) mediated the ubiquitination of RPS3 at K214, leading to the degradation of RPS3. These alterations promoted the cytoplasmic localization of RPS3 to inhibit the activation of NF-κB signaling, thereby enhancing the cisplatin sensitivity of EOC cells (40). Another study demonstrated that the deubiquitinase USP35 regulated the efficacy of cisplatin in OC (39). In detail, the expression of USP35 was significantly increased in OC, and knockdown of USP35 reduced the tumor burden in mice. When mice were treated with cisplatin, USP35 knockdown significantly reduced the tumor weight and dissemination. Further investigation revealed that USP35 removed the ubiquitin chains from stimulator of interferon gene (STING) to attenuate interferon signaling, thereby reducing the cisplatin-induced anti-tumor responses (39). As such, ubiquitination is important for regulating efficacy of OC treatment and is therefore a potential target for drug development.

### 2.2 Phosphorylation

**Figure 3:**
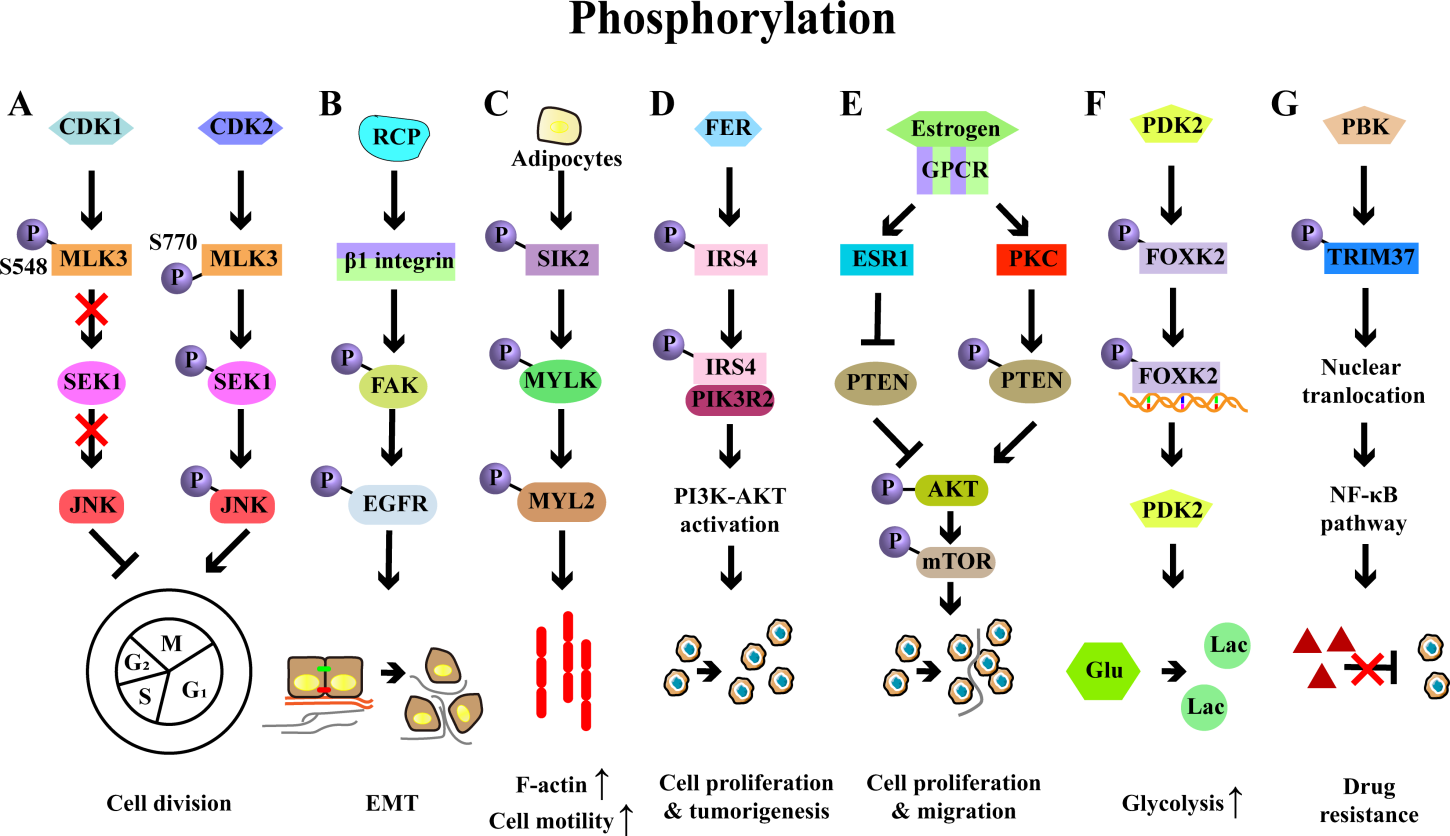
Examples of protein phosphorylation and their roles in ovarian cancer. **(A)** Phosphorylation of MLK3 by CDK1/2 on Ser548/Ser770 reduces or increases MLK3 activity, which influence the activation of SEK1-JNK pathway, thereby regulating the cell cycle of OC cells. **(B)** RCP stabilizes the integrin β1 and induces the phosphorylation of FAK-EGFR, leading to the OC invasion and EMT. **(C)** Adipocytes induces the phosphorylation of SIK2, which then phosphorylates MYLK and activates its downstream effector MYL2, ultimately promoting the motility and metastasis of OC cells. **(D)** FER-mediated phosphorylation of IRS4 enhances recruitment of PIK3R2/p85β and then activates PI3K-AKT signaling pathway, eventually resulting in cell proliferation and tumorigenesis. **(E)** Estrogen diminishes PTEN expression via the estrogen receptor 1 (ESR1) in OC cells and enhances the phosphorylation of PTEN by PKC, thereby promoting cell proliferation and migration via PI3K/AKT/mTOR signaling pathway. **(F)** PDK2-induced FOXK2 phosphorylation sustains glycolysis through a positive feedback manner in OC. **(G)** PBK interacts with TRIM37 to induce its phosphorylation and nuclear translocation, leading to the activation of NF-κB signaling and ultimately conferring drug resistance.

Phosphorylation, the most extensively characterized PTM, serves as a pivotal mechanism for regulating protein functions, including protein activities, subcellular localization, and signaling transduction (41). Serine (Ser), threonine (Thr), and tyrosine (Tyr) serve as primary residues for phosphorylation and dephosphorylation processes, mediated by kinases and phosphatases, respectively (42) Phosphorylation signaling is the most crucial pathway in cancers, influencing tumor growth, progression, and therapeutic responsiveness (12, 43). A quintessential example is the phosphoinositide 3-kinase-protein kinase B-mammalian target of rapamycin (PI3K/AKT/mTOR) signaling cascade, an important axis in cancer biology, widely targeted in therapeutic strategies for various cancers including breast, liver, and gastric cancers (44–46). In OC, increased expression of protein kinases such as doublecortin-like kinase 1 (DCLK1), nonreceptor tyrosine kinase feline sarcoma-related (FER), PDZ-binding kinase (PBK), CK2α, and cyclin-dependent kinase (CDK) has been reported, highlighting the pivotal role of phosphorylation signaling in the regulation of OC (**Figure 3**) (47–51).

#### 2.2.1 Phosphorylation in cancer progression

A key study has identified that phosphorylation of mixed lineage kinase 3 (MLK3), a member of the MAP3K family, modulates the division of OC cells through phosphorylation (52). Specifically, CDK1 promoted the phosphorylation of MLK3 on Ser548, which reduced the activity of MLK3 and inactivated SEK-JNK signaling, leading to a G2/M arrest in OC cells. Conversely, CDK2 phosphorylated MLK3 at Ser770, which enhanced its activity and activated SEK-JNK signaling, ultimately promoting cell cycle progression (52). Focal adhesion kinase (FAK), a key signaling factor, accelerated OC progression by orchestrating immunosuppression (53). Rab coupling protein (RCP), a pivotal player in cancer invasion, promoted β1 integrin expression, which led to the phosphorylation of FAK and subsequent activation of the epidermal growth factor receptor (EGFR), thereby driving OC cell invasion (54). Serine/threonine-protein kinase 2 (SIK2) has been identified to promote the motility, migration, and metastasis of OC cells both *in vitro* and *in vivo* (55). In detail, SIK2 induced the phosphorylation of myosin light chain kinase, smooth muscle (MYLK), which then phosphorylated and activated myosin light chain 2 (MYL2), ultimately facilitating cell motility and OC metastasis. Clinically, high co‐expression of SIK2 and MYLK‐pS343 was associated with the poor prognosis of OC (55).

Increasing evidence has demonstrated that common signaling molecules such as PI3K, AKT, and STAT1/3 are key regulators in OC progression (56, 57). For instance, the upregulation of insulin receptor substrate 4 (IRS4) was inversely correlated with prognosis of OC patients, and IRS4 was crucial for the activation of PI3K-AKT signaling and subsequent cell proliferation. Specifically, FER bound to and phosphorylated the IRS4, which promoted its recruitment of PIK3R2/p85β, resulting in the activation of PI3K-AKT signaling and the OC development (57). Phosphatase and tensin homolog (PTEN), a negative regulator of PI3K-AKT signaling, played an important role in estrogen-induced proliferation and migration of OC cells. PTEN knockdown enhanced the estrogen-induced proliferation of OC cells, while inhibition of PTEN phosphorylation curtailed the proliferation of OC cells, accompanied by the decreased phosphorylation of AKT and mTOR (58). Alterations of STAT1/STAT3 phosphorylation have also been reported to influence the anti-tumor T cell response in OC (56). In a mouse model of OC, the loss of tissue transglutaminase (TG2, an enzyme highly expressed in cancer cells) reduced the tumor burden in mice and decreased levels of myeloid cells (such as MDSCs and TAMs) in the peritoneal TME, while promoting the accumulation of CD8^+^ T cells in ascites and enhancing the cytotoxic function of CD8^+^ T cells (56). Mechanistically, TG2 deficiency increased levels of p-STAT1 and decreased levels of p-STAT3, thereby supporting the differentiation and function of cytotoxic T cells (56).

Phosphorylation also plays an important role in cancer metabolism. A recent study has demonstrated that the phosphorylation of transcription factor-forkhead box K2 (FOXK2) sustains glycolysis in OC cells (59). FOXK2, which was highly expressed and associated with the poor prognosis, regulated glycolysis in OC. Inhibition of FOXK2 reduced the OC growth, accompanied by the reduced levels of glycolysis (59). This regulation was mediated by phosphoinositide-dependent kinase 2 (PDK2, a key regulator of glycolysis and oxidative phosphorylation), which phosphorylated FOXK2, enhancing its transcriptional activity and upregulating glycolytic genes (including PDK2), thereby forming a positive feedback of glycolysis to support OC development (59).

#### 2.2.2 Phosphorylation in drug-resistance

Fibroblast growth factor receptor 3 (FGFR3) has been identified as a promoter of cisplatin resistance through the phosphorylation of the epidermal growth factor receptor (EGFR), which subsequently activates PI3K-AKT signaling pathways (60). Cofilin-1 phosphorylation has been reported to enhance paclitaxel resistance of OC cells via inhibiting their apoptosis, with this effect mediated by the knockdown of slingshot-1 (SSH-1) (61). PARP inhibitors (PARPi) are optional for OC treatment, however, PBK has been revealed to drive olaparib (Ola) resistance in OC cells. Mechanistically, PBK interacted with tripartite motif-containing 37 (TRIM37) to induce its phosphorylation and nuclear translocation, which further activated the NF-κB pathway and finally conferring resistance to PARPi in OC (62).

### 2.3 Glycosylation

Protein glycosylation, which includes *N-*glycosylation and *O-*glycosylation, involves the covalent attachment of glycans to proteins (63). Hypoxia may drive the alterations of glycosylation profiles in OC, and glycosylation has been revealed to participate in the OC pathogenesis and drug-resistance (64–66). The representative glycosylated protein is CA125, a high molecular weight MUC16 member containing 249 potential *N-*glycosylation and over 3700 *O*-glycosylation sites (67). Functionally, CA125 can interact with galectin-1 and mesothelin, which contributes to OC development by enhancing cell adhesion and accelerating metastasis (68–70). Clinically, CA125 has been utilized for OC diagnosis and for monitoring therapeutic effects and prognosis (68).

Human epididymis protein 4 (HE4), another biomarker for OC, is a secretory *N-*linked glycoprotein that has been introduced in clinical use (71). A previous study has demonstrated that the overall sensitivity and specificity of HE4 for OC diagnosis are 79% and 93%, respectively (71). Compared to CA125 and HE4 alone, the combination of CA125 and HE4 exhibited the highest AUC value of 0.847 and 0.927 (premenopause and postmenopause) (72). The risk of ovarian malignancy algorithm (ROMA), which included CA125, HE4, and menopausal status, demonstrated excellent diagnostic performance with an AUC of 0.935 in postmenopausal patients, accompanied by the sensitivity of 0.929 and specificity of 0.800 (72). Thus, CA125, HE4, and ROMA should be used complementarily for accurate diagnosis of OC. Additionally, HE4 has been implicated in establishing a suppressive TME and promoting chemoresistance (73, 74). Rats injected with HE4^hi^ ovarian cancer cells exhibited elevated levels of myeloid-recruiting and differentiation factors in the ascites, accompanied by an influx of M2 macrophages and enhanced arginase 1 production. Moreover, the activation of CTLs in ascites was significantly reduced. HE4 upregulated the expression of PD-L1 on tumor cells and macrophages, indicating HE4 was a mediator of immunosuppression (74). Another study demonstrated that HE4-overexpressing OC cells displayed increased resistance to cisplatin and paclitaxel, while knockdown of HE4 reversed these effects. MAPK signaling-mediated apoptosis and alterations in tubulin levels or stability were involved in this chemoresistance (73).

A previous study has demonstrated that IgG-specific glycosylation profiles are highly capable of discriminating between EOC patients and controls. This study revealed that a glycopeptide from IgG1 with glycan moiety H_5_N_5_F_1_ exhibited best classification performance (75). A latter study also confirmed this observation, which demonstrated that glycosylation of IgG1 was most strongly affected in EOC, as indicated by the highest number of significant differences between healthy controls and EOC patients. However, less pronounced pattern of IgG2 glycosylation alterations was observed. Moreover, IgG1 and IgG3 agalactosylation exhibited strong associations with CA125, suggesting that specific glycosylation changes in IgG subclasses might serve as biomarkers for OC diagnosis (76).

**Figure 4:**
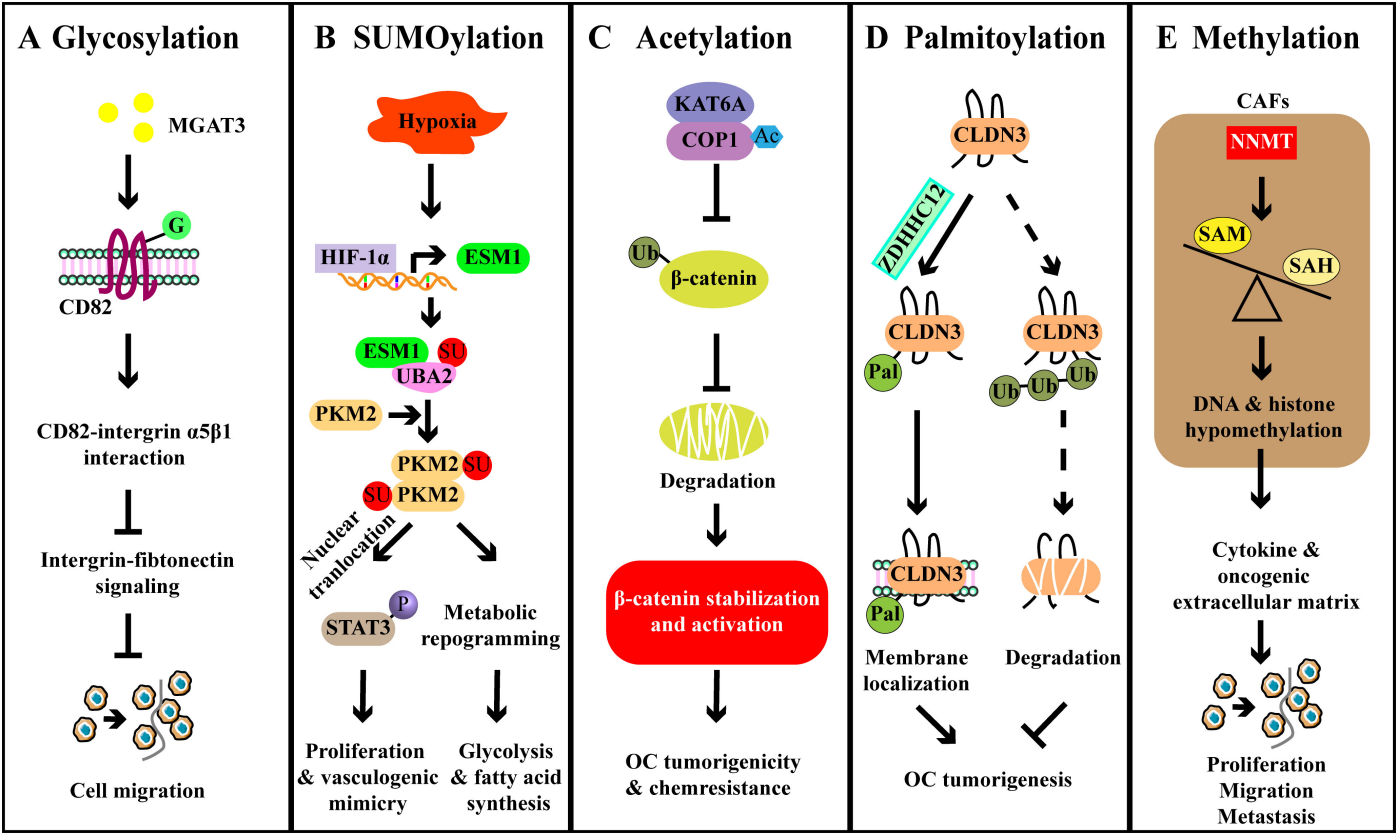
Examples of other PTMs and their roles in ovarian cancer. **(A)** MGAT3-mediated CD82 glycosylation binds to integrin α5β1 and disrupts the integrin-fibronectin signaling, thereby inhibiting OC cell metastasis. **(B)** HIF-1α upregulates the expression of ESM1, and then ESM1 promotes the activation of UBA2, leading to the SUMOylation of PKM2. PKM2 SUMOylation promotes the metabolic reprogramming, enhancing the fatty acid synthesis. PKM2 SUMOylation also enhances the nuclear localization of the PKM2 dimer and promotes the activation of STAT3, thereby facilitating downstream oncogene expression to promote vascular mimicry. **(C)** KAT6A acetylates COP1 to impair its function as an E3 ligase and contributes to the stabilization and activation of β-catenin, thereby promoting OC tumorigenicity and chemoresistance. **(D)** ZDHHC12-mediated CLDN3 palmitoylation promotes the cell membrane localization of CLDN3 and reduces its degradation, leading to the tumorigenesis. **(E)** Stromal NNMT regulates DNA-histone methylation through the metabolic program, leading to oncogenic cytokines and extracellular matrix expression in stromal cells that promote OC cell proliferation, migration, and metastasis.

Altered expression of integrin and tetraspanin is critical for cancer cell migration and invasion, and aberrant glycosylation of these proteins has been reported in EOC (77, 78). Glycovariants of ITGα3 (ITGα3^STn^) exhibited a higher value of AUC than ITGα3^IA^ in ovarian cyst fluid, and the combined ITGα3-based assays that include ITGα3^IA^ and ITGα3^STn^ identified 49 out of 55 malignant and borderline cases, while none of the 22 benign and healthy cysts were detected, suggesting the potential of ITGα3 glycosylation as diagnostic markers in EOC (77). Glycosylation of CD82 has been found to suppress the metastasis of OC by inhibiting the integrin signaling (78). CD82 was glycosylated in OC cells, but the glycosylation of CD82 might be impaired in metastatic OC. Further investigation revealed that glycosylation at N157 site was critical for the inhibition of OC cell migration *in vitro*. *In vivo*, CD82 glycosylation at N157 site inhibited metastasis formation (78). Mechanistically, there was an interaction between glycosylated CD82 and ITGα5β1, this interaction impaired the integrin-fibronectin interaction and then inhibited the cell motility and migration. Furthermore, mannoside acetylglucosaminyltransferase 3 (MGAT3) was responsible for CD82 glycosylation, because knockdown of MGAT3 significantly reduced the glycosylation of CD82. Collectively, MGAT3-mediated glycosylation of CD82 at N157 suppressed OC metastasis by downregulating the integrin signaling (**Figure 4A**) (78). Taken together, these findings underscore the potential of protein glycosylation not only for disease monitoring but also as a therapeutic target in OC.

### 2.4 SUMOylation

SUMOylation is a ubiquitination-like PTM that primarily regulates protein localization and function (79). The small ubiquitin-related modifier (SUMO) protein is evolutionarily conserved across species, with four SUMO genes identified in mammals, namely SUMO1-4 (80). A recent study revealed that monensin could suppress the proliferation and colony formation of OC cells (81). Monensin reduced the mitogen-activated protein kinase (MEK)-ERK signaling, which played a crucial role in epithelial-to-mesenchymal transition (EMT) (81). Mechanistically, monensin increased the SUMOylation of MEK1, thereby diminishing its activity. Removal of SUMOylation increased MEK1 activity, leading to the recovered viability and proliferation of OC cells (81). A previous study has shown that overexpression of ubiquitin-conjugating enzyme 9 (Ubc9, the SUMO E2 ligase) promotes the proliferation of OC cells, whereas Ubc9-silenced cells exhibit reduced proliferation. However, this effect is associated with the phosphorylation of AKT and activation of PI3K-AKT pathway, not SUMOylation (82). Besides, SUMOylation also has been implicated in metabolic adaption (83). This study suggested that HIF-1α-induced the upregulation of endothelial cell specific molecule 1 (ESM1) activated the SUMO1 through the ubiquitin-like modifier-activating enzyme 2 (UBA2), leading to PKM2 SUMOylation and dimerization. These alterations contributed to the nuclear localization of PKM2 and subsequent the phosphorylation of STAT3, ultimately promoting OC glycolysis and vasculogenic mimicry (**Figure 4B**) (83). However, further studies are required to explore the broader roles of SUMOylation in OC, including its implications in cell apoptosis, immune responses, and chemoresistance.

### 2.5 Acetylation

Protein acetylation is a kind of reversible PTM that significantly impacts the protein stability, localization, and interaction (84, 85). This process can be regulated through specific enzymes, including lysine acetyltransferases (KATs) and lysine deacetylases (KDACs) (84). Most KATs can be classified into families including general control non-depressible 5 (GCN5), p300, and members MOZ, Ybf2/Sas3, Sas2, and Tip60 (MYST), while most KDACs are classified into the classical Zn_2_^+^-dependent histone deacetylases (HDACs) and the NAD^+^-dependent sirtuin deacetylases (84).

KAT6A, a MYST-type acetyltransferase identified as an oncogene in OC, was associated with the poor prognosis of OC patients (23). *In vivo*, knockdown of KAT6A not only inhibited cancer growth but also impaired the ability of cancer cells to form metastasis. Similar effects were observed *in vitro*, as evidenced by decreased cell proliferation and colony formation after depleting KAT6A (23). Mechanistically, KAT6A associated with COP1 and acetylated the K294 residue of COP1, which impaired the activity of COP1 as an E3 ubiquitin ligase to catalyze the ubiquitination of β-catenin. Finally, the accumulation and activation of β-catenin contributed to the growth, invasion, and chemoresistance of OC (**Figure 4C**) (23). As a tumor-suppressor, aplasia Ras homolog member I (ARHI) inhibited cell growth and induced apoptosis of OC cells. However, ARHI was typically downregulated in most OC cells (86). It has been demonstrated that acetylated STAT3 can interact with the ARHI promoter and recruit DNA methyl transferase 1 (DNMT1), leading to the promoter methylation and consequent ARHI downregulation, thereby promoting the proliferation of OC cells (87). High-mobility group protein box-1 (HMGB1) is associated with the chemoresistance of tumor cells, and its expression is considered to be a promising biomarker for OC (88). In OC cells, HMGB1 was found to be regulated by sirtuin 1 (SIRT1) (89). In detail, the overexpression or knockdown of SIRT1 decreased or enhanced the expression and acetylation of HMGB1, respectively. Functionally, SIRT1 overexpression inhibited the migration and angiogenesis of OC (89).

Histone acetylation plays a crucial role in the regulation of gene expression and is associated with the progression of cancer (90). Histone acetyltransferase (HAT) binding to ORC-1 (HBO1), a member of MYST family, associated with JADE and subsequently acetylated histone H4, which was crucial for the expression of ovarian cancer oncogene and mechanical signaling factor YAP1 (91). Conversely, HBO1 deficiency increased cell viability and significantly reduced cell membrane elasticity (91). Importantly, the histone acetylation-related signature has been proven to be a valuable tool for predicting the prognosis of OC (92, 93). Based on the TCGA database, *HDAC1, HDAC2, HDAC4, HDAC10, HDAC11, KAT7, ELP3, KIAA 2026, SP140*, and *SIRT5* were selected as a histone acetylation modulator-related signature (93). The last four factors indicated a good prognosis, while the others were associated with the poor prognosis. In addition, *HDAC1, HDAC10*, and *KAT7* have been verified as independent prognostic factors for OC (93). Similarly, another study identified a prognostic gene signature comprising *SIRT5*, bromodomain-containing protein 4 (*BRD4*), *OGA, SIRT2, HDAC4, NCOA3, HDAC1*, and *HDAC11* (92). Taken together, these findings highlight the potential of targeting acetylation to regulate OC development and predict patient outcomes.

### 2.6 Palmitoylation

Protein palmitoylation, an important form of PTMs, can be classified into two forms: reversible (S-palmitoylation) or irreversible (N-palmitoylation) (94). Palmitoylation is primarily catalyzed by the zinc finger DHHC-type containing (ZDHHC) family of palmitoyl transferases (PATs), while depalmitoylation is mediated by depalmitoylases (95).

A recent study has reported that the tumorigenesis of OC was significantly inhibited by the knockdown of ZDHHC12 (96). Mechanistically, ZDHHC12 mediated the S-palmitoylation of membrane protein claudin-3 (CLDN3) at three juxtamembrane cysteine residues, which promoted cell membrane localization of CLDN3, ultimately determining malignant progression (**Figure 4D**). The knockdown of ZDHHC12 not only inhibited the stability of CLDN3 but also restricted cancer progression (96). Similarly, ZDHHC18 has also been reported to enhance tumor growth (97). ZDHHC18 interacted with malate dehydrogenase 2 (MDH2) and mediated its palmitoylation at C138 residue, which activated mitochondrial respiration and promoted the growth of OC (97). Moreover, the knockdown of MDH2 led to the suppression of mitochondrial respiration and OC cell proliferation both *in vitro* and *in vivo* (97). In high-grade serous OC (HGSOC) patients, increased levels of ZDHHC18 and palmitoylated MDH2 have been detected (97). As such, palmitoylation is important for OC progression and might be a promising pharmaceutical target for future treatments.

### 2.7 Methylation

DNA methylation plays a diverse role in regulating gene expression across various biological processes. Recently, protein methylation has been demonstrated in the regulation of OC development, especially histone methylation (98–101). Elevated levels of C/EBPβ have been associated with the poor prognosis in OC patients, possibly due to enhanced cisplatin resistance (101). Mechanistically, C/EBPβ interacted with DOT1L to induce H3K79 methylation of multiple drug-resistance genes, ultimately resulting in cisplatin resistance (101). Similarly, histone-lysine N-methyltransferase 2D (KMT2D)-mediated H3K4 methylation and G9a-mediated H3K9 methylation also participated in the pathogenesis and metastasis of OC (98, 100). Notably, the role of demethylation of histones in cancer-associated fibroblasts (CAFs) has been implicated in OC progression (102). The expression of nicotinamide N-methyltransferase (NNMT) in CAFs led to a reduction in S-adenosyl methionine (SAM)/S-adenosyl-L-homocysteine (SAH) ratio, resulting in the decreased levels of H3K27 and H3K4 trimethylation (102). This reduction enhanced the tumor-supportive functions of CAF, including the secretion of cytokines and oncogenic extracellular matrix, thereby promoting OC progression (**Figure 4E**) (102). In addition, non-histone methylation has also been documented in the regulation of OC development. CARM1, for example, induced the methylation of BAF155, downregulating the expression of EZH2/BAF155 target tumor suppressor genes (99). However, compared to the histone methylation, studies on non-histone protein methylation still need to go a step further.

## 3 PTMs: potential targets for ovarian cancer treatment

As described above, the dysregulation of PTMs is associated with the proliferation, migration, and chemoresistance of ovarian cancer cells, thereby impacting cancer progression and the efficacy of treatments. Therefore, the development of drugs targeting PTMs holds promising therapeutic potential for OC. In this section, we mainly introduce the modulators of ubiquitination, SUMOylation, phosphorylation, and acetylation, with modulators targeting other PTMs have been summarized in **Table 2**.

**Table 2:** Modulators of protein PTMs and their effects in ovarian cancer.

Modulators	Target protein	Dosages	Function	Therapeutic effects	References
Dihydrotanshinone I	Nrf2	1.6-16 μM *in vitro* 10 or 20 mg/kg *in vivo*	Promotes Keap1-mediated Nrf2 ubiquitination	Inhibits the apoptosis of OC cells	(103)
Metformin	TRAF2	20 mM *in vitro*	Suppresses TRIM37-induced TRAF2 ubiquitination	Inhibits the proliferation and invasion of OC cells	(104)
O-phenanthroline	PKM2	1.25-10 μM *in vitro* 25 mg/kg *in vivo*	Suppresses PSMD14-mediated cleavage of K63-linked ubiquitination on PKM2	Reduces the ability of growth, migration and invasion of OC	(37)
Monensin	MEK1	0.2, 1 and 5 μM *in vitro* 8 or 16 mg/kg *in vivo*	Promotes MEK1 SUMOylation	Inhibits cell proliferation, invasion, and malignant transformation	(81)
MOTS-c	LARS1	20 or 30 μM *in vitro* 20 mg/kg *in vivo*	Attenuates USP7-mediated LARS1 deubiquitination	Inhibits the proliferation, migration, and invasion of OC cells	(105)
SAHA	H2A, H2B, H3, and H4	7.5 μM *in vitro*	Promotes histone acetylation	Induces the death of OC cells	(110)
WM-1119	COP1	25 μM *in vitro* 60 mg/kg *in vivo*	Suppresses KAT6A-mediated COP1 acetylation	Enhances the antitumor activity of cisplatin	(23)
JM7	TEAD	2 μM *in vitro*	Suppresses TEAD palmitoylation	Inhibits the proliferation, colony forming and migration of OC cells	(133)
Matrine	Cancer associated phosphorylation signaling	1.0 or 2.0 mg/mL *in vitro* 100 mg/kg *in vivo*	Suppresses their phosphorylation	Induces the apoptosis and autophagy of OC cells	(106)
Curcumin	FAK	20 μM *in vitro*	Suppresses Rab coupling protein-induced FAK phosphorylation	Inhibits the invasion of OC cells	(54)
Berberine	FAK	5 μmol/L *in vitro*	Suppresses FAK phosphorylation	Inhibits the chemotherapy-induced repopulation	(107)
Difluoromethylornithine	JNK	10-100 μM *in vitro*	Promotes JNK phosphorylation	Induces the apoptosis of OC cells	(108)
MLN4924	Cullin	0.1-1 μM *in vitro*	Disrupts cullin NEDDylation	Induces the apoptosis of OC cells	(130, 134, 135)
GSNO	STAT3	0.1-1 mM *in vitro* 1 mg/kg *in vivo*	Promotes STAT3 nitrosylation	Abrogates the growth of OC cells	(136)
5-amino-1-methylquinolin-1-ium	Nicotinamide N-methyltransferase	10 μM *in vitro* 20 mg/kg *in vivo*	Promotes Histone H3 (K4 and K27) methylation	Reduces the proliferation of OC cells	(102)
UNC0638 and 5-Aza-CdR	G9A	25-4800 nM *in vitro* 100-2000 nM *in vitro*	Suppresses Histone H3 (K9) methylation	Promotes the death of OC cells	(137)
GSK126	EZH2	10 μM *in vitro* 50 mg/kg *in vivo*	Disrupts CARM1-enhanced EZH2-mediated silencing of tumor suppressor genes	Inhibits the growth of OC	(99)

### 3.1 Modulators of ubiquitination and SUMOylation

For ubiquitination and SUMOylation, dihydrotanshinone I (DHT), metformin, and monensin have been found to hinder the progression of OC by targeting these modifications (81, 103, 104). DHT decreased the cell viability of multiple OC cell lines and induced their apoptosis by activating oxidative stress (103). In detail, DHT promoted the interaction between Kelch-like ECH-associated protein (Keap1) and NF-E2-related factor 2 (Nrf2), which enhanced the Nrf2 ubiquitination and degradation, leading to the ROS accumulation and thereby enhancing the anti-tumor effects (103). Metformin inhibited the proliferation and invasion of OC cells by suppressing TRIM37-induced ubiquitination of TRAF2, accompanied by the reduced activation of NF-κB signaling (104). As previously stated, PSMD14 facilitated the progression of OC through the cleavage of K63-linked ubiquitination on PKM2, which could be efficiently reversed by O‐phenanthroline (OPA, an inhibitor of PSMD14) (37).

Mitochondrial ORF of the 12S rRNA Type-C (MOTS-c), a short peptide encoded by mitochondrial 12S rRNA, has been revealed to prevent the OC progression by regulating protein deubiquitination (105). MOTS-c underwent a reduction in OC, which was associated with the poor prognosis. *In vitro*, MOTS-c treatment significantly suppressed the proliferation of OC cells, accompanied by the increased apoptosis rate. Further investigation revealed that MOTS-c interacted with LARS1 (a cancer-promoting factor in OC), leading to its K48-linked polyubiquitination on the K243 residue and subsequent degradation (105). Mechanistically, MOTS-c competed with USP7 for binding LARS1, reducing USP7-mediated deubiquitination and thereby leading to LARS1 degradation (105). Importantly, MOTS-c effectively inhibited the OC growth without toxicity in the xenograft model, suggesting its potential as a viable target for OC diagnosis and treatment (105).

### 3.2 Modulators of phosphorylation

Matrine, a natural alkaloid, not only suppressed the proliferation, migration, and invasion of OC cells but also induced apoptosis and autophagy (106). These effects were dependent on the suppression of cancer-associated phosphorylation signaling pathways, including ERK1/2, MEK1/2, PI3K, AKT, mTOR, FAK, RhoA, VEGFR2, and Tie2 (106). Curcumin and berberine are two compounds that prevent OC progression by targeting FAK phosphorylation (54, 107). Curcumin disrupted the stabilization of β1 integrin to limit FAK and EGFR signaling activation, whereas berberine inhibited the production of prostaglandin E2 (PGE2) and PGE2-elicited FAK phosphorylation, thereby preventing the progression and repopulation of OC (54, 107). Difluoromethylornithine (DFMO), an inhibitor of ornithine decarboxylase, induced the apoptosis of OC cells through AP-1 via JNK phosphorylation. Moreover, DFMO could enhance the effect of cisplatin (108).

### 3.3 Modulators of acetylation

WM-1119, an inhibitor of KAT6A, has been discovered to decrease the acetylation of COP1, subsequently facilitating the ubiquitination-mediated degradation of β-catenin and ultimately leading to the improvement of anti-tumor effects triggered by cisplatin (23). Suberoylanilide hydroxamic acid (SAHA) is a histone deacetylase inhibitor (HDACi) that inhibits the removal of acetyl group (109). SAHA induced cell death of A2780 cells and suppressed spheroid formation. Mechanistically, SAHA promoted the hyperacetylation of histones (including H2A, H2B, H3, and H4), thus reducing the levels of tumor progression-associated DNA methyl transferases/histone methyl transferases (DNMTs/HMTs) (110). These HADCis can be used alone or in combination with other drugs, such as azacitidine, carboplatin, as well as 5-Fluorouracil (5-FU).

## 4 Clinical applications of PTM modulators in OC

The efficacy of PTM modulators in clinical trials have been widely investigated, particularly for HDAC inhibitors and phosphorylation modulators (ClinicalTrials.gov). This section primarily introduces the application and efficacy of these modulators in treating OC (**Table 3**).

**Table 3:** Examples of PTM-targeting clinical trials for ovarian cancer.

Trial ID	Target	Intervention/Treatment	Phase	Conditions	Status
NCT00132067	HDAC	Vorinostat	Phase 2	Recurrent or persistent ovarian epithelial cancer	Completed
NCT00976183	HDAC	Vorinostat/carboplatin/paclitaxel	Phase 1/2	Advanced stage OC	Terminated
NCT00910000	HDAC	Vorinostat/carboplatin/gemcitabine	Phase 1b/2	Recurrent, platinum-sensitive OC	Terminated
NCT01653912	AKT	Afuresertib/carboplatin/paclitaxel	Phase 1/2	Platinum-resistant OC	Completed
NCT04374630	AKT	Afuresertib/paclitaxel	Phase 2	Platinum-resistant OC	Completed
NCT02203513	CHK1	Prexasertib	Phase 2	*BRCA1/2* mutation-associated OC, HGSOC	Terminated
NCT02482311	WEE1	Adavosertib	Phase 1b	Advanced stage OC	Completed
NCT02151292	WEE1	Adavosertib/gemcitabine	Phase 2	Recurrent, platinum-resistant OC	Active, not recruiting
NCT01164995	WEE1	Adavosertib/carboplatin	Phase 2	*Tp53* mutated refractory and resistant OC	Completed
NCT05128825	WEE1	Azenosertib	Phase 2	HGSOC	Active, not recruiting
NCT03639246	AXL	Batiraxcept/pegylated liposomal doxorubicin/paclitaxel	Phase 1b/2	Recurrent, platinum-resistant OC	Completed
NCT04729608	AXL	Batiraxcept/paclitaxel	Phase 3	Recurrent, platinum-resistant OC	Terminated

### 4.1 HDAC inhibitors

Several HDACi has been tested in clinical trials, including vorionostat and belinostat (111, 112). A phase 2 trial was conducted to study how vorinostat performed in treating patients with recurrent or persistent epithelial ovarian or primary peritoneal carcinoma (NCT00132067). According to the published data, two participants (total twenty-seven) survived progression-free over 6 months, with only two instances of grade 4 toxicities reported. These results indicated that while vorinostat was well-tolerated, it showed minimal activity as a monotherapy in these patients, highlighting the potential need for combination therapies (113). In another study, patients with advanced stage OC received vorinostat in conjunction with paclitaxel and carboplatin (NCT00976183). The total response rate was reported at 50%, with nine patients (56.3%) developing grade 3/4 neutropenia and two patients (12.5%) experiencing thrombocytopenia, respectively. Regrettably, significant gastrointestinal events resulted in the closure of this trial (114). A subsequent trial combining carboplatin, gemcitabine, and vorinostat aimed to assess their efficacy in patients with recurrent, platinum-sensitive OC (NCT00910000). Out of fifteen patients, seven were evaluable for response according to the response evaluation criteria in solid tumor (RECIST) assessment. Among seven patients, five achieved confirmed partial responses (PRs) and one was unconfirmed, accompanied by one patient with stable disease. These evidence suggested the efficacy of the combination therapy in relapsed platinum-sensitive OC, but the trial was terminated early due to the unacceptable hematologic toxicities (112).

### 4.2 AKT inhibitors

As we described above, the dysregulation of the PI3K-AKT signaling contributes the OC progression. A phase 1/2 study (NCT01653912) evaluated the efficacy of AKT inhibitor afuresertib in combination with paclitaxel and carboplatin in platinum-resistant OC, with twenty-nine patients were enrolled into Part I (dose escalation) and thirty patients into Part II (dose expansion) (24). The confirmed objective response rate (ORR) was 24% in intention-to-treat (ITT) population of Part I, and the confirmed ORR per RECIST was 32% in the ITT population of Part II. The most common adverse effects included nausea, diarrhea, fatigue, and alopecia in both groups. These findings demonstrated the efficacy of afuresertib/carboplatin/paclitaxel in treating recurrent platinum-resistant OC (24). Thus, the combination of afuresertib plus weekly paclitaxel might represent a clinically meaningful step for platinum-resistant OC, with the results still need to be revealed (NCT04374630).

### 4.3 ATR/CHK1 inhibitors

Ataxia-telangiectasia-mutated-and-Rad3-related kinase (ATR), a member of PI3K-related kinase (PIKK) family that regulates cell cycle through activating checkpoint kinase 1 (CHK1), is an attractive therapeutic target. Ceralasertib-mediated ATR inhibition plus Ola showed an ORR of 8.3% and a clinical benefit rate of 62.5%, suggesting the preliminary activity in patients with *BRCA*-mutated, PARPi-resistant HGSOC (115). CHK1 facilitates the cell with sustained DNA damage to death when it is inhibited. Prexasertib, a CHK1 inhibitor (CHK1i), was evaluated in twenty-eight patients with *BRCA* wild-type recurrent HGSOC as part of a phase 2 study (NCT02203513). This study demonstrated that 33% of twenty-four evaluable patients achieved PRs, and the PR rate in the ITT population was 29% (116). In May 2023, the US Food and Drug Administration granted fast track designations to prexasertib monotherapy for the treatment of patients with platinum-resistant OC who were positive for predicted sensitivity to the agent. A recent study reported the activity of prexasertib in platinum-resistant HGSOC with measurable and biopsiable disease (cohort 5), or without biopsiable disease (cohort 6) (117). Among the thirty-nine evaluable patients, the ORR in cohort 5 and cohort 6 were 33.3% and 28.5%, respectively. Toxicity was manageable, and hematological toxicities were the most common grade 3 or 4 treatment-related adverse events (117). Additionally, preclinical investigation revealed that combining polymerase alpha 1 (POLA1) inhibition and CHK1i could synergistically inhibit the growth of *BRCA* wild-type, platinum-resistant HGSOC cell lines, indicating their therapeutic potential to overcome CHK1i resistance (117).

### 4.4 WEE1 inhibitors

WEE1, similar to ATR/CHK1, is a protein kinase that can regulate the cell cycle, and there are WEE1 inhibitors have been evaluated in clinical trials (118). A phase 1b study of adavosertib monotherapy reported an ORR of 3.3% and a progression-free survival (PFS) of 3.9 months in *BRCA*-mutated group (NCT02482311) (119). Then, the efficacy of adavosertib plus gemcitabine was evaluated in patients with recurrent, platinum-resistant EOC (NCT02151292). Patients received adavosertib plus gemcitabine showed extended PFS than the control group (median month 4.6 vs 3.0), without treatment-related fatalities (25). The combination of adavosertib and carboplatin was safe and effective in patients with *TP53*-mutated OC (NCT01164995) (120). Subsequent investigation revealed that the ORR was 41% in the evaluable patients, accompanied by the PFS of 5.6 months. However, bone marrow toxicity was the most common adverse event leading to the dose reductions and dose delays (121).

### 4.5 AXL inhibitors

AXL is a receptor tyrosine kinase that belongs to the TYRO3, AXL, and MERTK (TAM) family, which controls the tumor growth and EMT upon the binding of growth arrest-specific protein 6 (GAS6) (122). Batiraxcept is a potent and specific AXL decoy protein that binds to GAS6 and inhibits its interaction with AXL, thereby reducing downstream signaling (such as JAK-STAT and PI3K-AKT signaling) (123). In a phase 1b study involving patients with platinum-resistant OC, batiraxcept combined with paclitaxel achieved an ORR of 34.8%, with a median PFS of 3.1 months and overall survival of 10.3 months (NCT03639246) (123). The combination was well-tolerated, with no dose-limiting toxicities observed (123). Later, a phase 3 clinical trial was performed to compare this combination with the paclitaxel monotherapy in platinum-resistant HGSOC, but the trial was terminated as no significant differences in median PFS and OS between treatment arms (NCT04729608).

Overall, PTM modulators are promising therapies (monotherapy or combination therapy) for the treatment of ovarian cancer in that such modulators potentially regulate the critical proteins for cancer progression. However, adverse events such as hematologic toxicities are concerns remain to be improved.

## 5 Conclusions and future perspectives

Hitherto, it is clear that PTMs profoundly influence protein function and regulate disease progression (124, 125). In recent years, there has been an obvious increase in interest and understanding regarding the role of PTMs in cancer. Although there is still much to be investigated, it is promising to target PTMs for treatments given their profound impacts on oncogenesis.

With the deepening of research, the importance of protein PTMs in the regulation of OC progression has been gradually revealed and recognized. As previously discussed, PTMs regulate multiple aspects of OC, including oncogenic signaling, tumorigenic cytokines, autophagy, cell adhesion, and metabolic adaptation (20, 23, 59, 78). For instance, ZDHHC12-mediated CLDN3 palmitoylation contributes to the plasma membrane localization and protein stability of CLDN3, thereby determining OC progression (96). The ubiquitination of BECN1 and phosphorylation of PKM2 facilitates the OC progression by regulating autophagic flux and aerobic glycolysis, respectively (22, 59). Interestingly, there is also mutual influence between different PTMs. Protein arginine methyltransferase 1 (PRMT1)-induced methylation of BRD4 promoted the OC invasion through regulating the phosphorylation of BRD4 (126). Although the effects of PTMs on OC have been extensively explored, the underlying mechanisms that drive PTMs are still elusive. Hypoxia may be a factor driving PTMs, which has been revealed to alter *N-*glycosylation profiles and PKM2 SUMOylation in OC cells, thereby regulating OC progression (66, 83).

Therapies targeting PTMs have gained importance in the treatment of ovarian cancer due to their crucial functions. As described above, PTM modulators such as histone deacetylase inhibitors, dihydrotanshinone I, metformin, matrine, and monensin have shown potential in suppressing OC progression through the regulation of PTMs (81, 103, 104, 106, 108, 127). For instance, curcumin and berberine have been reported to inhibit OC development by suppressing the phosphorylation of FAK (54, 107). Clinical trials of PTM modulators, including AKT inhibitors, CHK inhibitors, and WEE1 inhibitors have been conducted and exhibited potential efficacy in treating OC (24, 25, 117). As presented by the results of clinical trials, co-administration of PTM modulators with existing drugs seem to be promising. Importantly, nanotechnology can be used for co-administration. A tumor-targeting peptide TMTP1 modified MPDA-based nano-drug delivery system (TPNPs) has been designed to co-deliver of adavosertib and Ola, which has shown therapeutic effects in OC models (128). Therefore, the following points remain further investigation: 1) the mechanisms driving the occurrence of PTMs, 2) novel sites and modulators of protein PTMs, 3) combination therapy with PTM modulators and other therapeutic strategies, 4) deliver system to improve the efficacy of modulators. Here, it is worth mentioning that a challenge will be the identification of PTMs and their corresponding enzymatic activities that are specific to particular cell types or lineages, in order to avert toxicities in non-target tissues. With the current research foundation and future research avenues, we believe that targeting PTMs holds the potential to offer novel therapeutic options for OC and other malignancies in the future.

## Glossary

OC: ovarian cancer
PTM: post-translational modification
TME: tumor microenvironment
CA125: carbohydrate antigen 125
FBXO: F-box only protein
CUL3: cullin 3
KAT6A: lysine acetyltransferase 6A
DUBs: deubiquitinases
SUN2: SAD1/UNC84 domain protein-2
RNASET2: ribonuclease T2
hnRNPL: heterogeneous nuclear ribonucleoprotein L
SMURF1: Smad ubiquitination regulatory factor 1
ARHGAP26: Rho GTPase-activating protein 26
GPBAR1: G-protein-coupled bile acid receptor-1
SOC: serous ovarian cancer
HIF-1α: hypoxia-inducible factor-1α
TRPM7: transient receptor potential 7
PSMD14: 26S proteasome non-ATPase regulatory subunit 14
PKM2: pyruvate kinase M2
OTUB2: Otubain 2
STING: synthase-stimulator of interferon genes
SIAH1: seven in absentia homolog 1
RPS3: ribosomal protein S3
EOC: epithelial OC
PI3K/AKT/mTOR: phosphoinositide 3-kinase-protein kinase B-mammalian target of rapamycin
FER: nonreceptor tyrosine kinase feline sarcoma-related
PBK: PDZ-binding kinase
CDK: cyclin-dependent kinase
MLK3: mixed lineage kinase 3
FAK: focal adhesion kinase
EGFR: epidermal growth factor receptor
SIK: serine/threonine-protein kinase
IRS4: insulin receptor substrate 4
PTEN: phosphatase and tensin homolog
FOXK2: forkhead box K2
PDK2: phosphoinositide-dependent kinase 2
FGFR3: fibroblast growth factor receptor 3
HE4: human epididymis protein 4
SUMO: small ubiquitin-related modifier
MEK: mitogen-activated protein kinase
ERK: extracellular signal-regulated kinase
EMT: epithelial-to-mesenchymal transition
Ubc9: ubiquitin-conjugating enzyme 9
K(H)AT: lysine (histone) acetyltransferase
K(H)DAC: lysine (histone) deacetylases
ARHI: aplasia Ras homolog member I
HMGB1: high-mobility group protein box-1
SIRT1: sirtuin 1
PATs: palmitoyl transferases
CLDN3: claudin-3
MDH2: malate dehydrogenase 2
HGSOC: high-grade serous ovarian cancer
TRIM37: tripartite motif-containing 37
CAFs: cancer-associated fibroblasts
Nrf2: NF-E2-related factor 2
TRAF2: tumor necrosis factor receptor-associated factor 2
SAHA: suberoylanilide hydroxamic acid
DNMTs/HMTs: DNA methyl transferases/histone methyl transferases
TEAD: transcriptional enhancer associate domain
PGE2: prostaglandin E2
